# Reducing door-to-wire time for ST-elevation myocardial infarction patients undergoing primary percutaneous coronary intervention by multidisciplinary collaboration: An observational study

**DOI:** 10.1097/MD.0000000000039297

**Published:** 2024-08-30

**Authors:** Xiaoru Zeng, Ling Chen, Eric Jou, Ayush Chandra, Guanglong Ma, Xiaodong Zheng, Junrong Tu, Jianguang Liang, Shengde Xie, Jiachao Liu, Francisco-Javier Roldan, Zhenzhang Li, Wanling Pan, Wanquan Li

**Affiliations:** a Internal Medicine-Cardiovascular Department, Foshan Sanshui District People’s Hospital, Foshan, Guangdong, China; b Medical Sciences Division, Oxford University Hospitals, University of Oxford, Oxford, United Kingdom; c Kellogg College, University of Oxford, Oxford, United Kingdom; d Department of Clinical Medicine, Tianjin Medical University, Tianjin, China; e Department of Emergency Medicine, Foshan Sanshui District People’s Hospital, Foshan, Guangdong, China; f Outpatient Department, National Institute of Cardiology, Mexico City, Mexico; g College of Mathematics and Systems Science, Guangdong Polytechnic Normal University, Guangzhou, China; h School of Basic Medical Sciences, Guangzhou Medical University, Guangzhou, China; i Department of Nursing, Foshan Sanshui District People’s Hospital, Foshan, Guangdong Province, China.

**Keywords:** coronary intervention, door to wire time, multidisciplinary, STEMI

## Abstract

The aim of this study is to reduce door-to-wire time for ST-elevation myocardial infarction patients undergoing primary percutaneous coronary intervention through multidisciplinary collaboration. Patients over the age of 18who visited the Foshan Sanshui District People’s Hospital between 2018 and 2019 and were diagnosed with STEMI were included in this study. Analyses were performed with patients segregated into a pre-intervention interim period (2018) and a post-intervention period (2019) based on the date of admission. Intervention measures for reducing door to wire time were fully implemented towards the end of the interim period. There were no significant differences in the baseline characteristics of the 2 groups. Median door to puncture time was reduced from 57.5 minutes in the interim period to 46.0 minutes (*P* < .001) in the post-intervention period. Similarly, median door to wire time was shortened from 88.0 minutes to 63.5 minutes (*P* < .001). During the interim period, 24% of patients had a door to wire time of <60 minutes, compared to 40.67% of patients in the post-intervention period (*P* = .002). Multidisciplinary collaboration is an important strategy to reduce door to wire time for patients with STEMI, and may be implemented in suitable centers to improve patient care.

## 1. Introduction

Acute myocardial infarction (AMI) remains to be one of the leading causes of morbidity and mortality worldwide despite contemporary advances in medicine.^[1]^ The incidence of AMI has increased over the past decades both globally and in China, and is projected to increase by a striking 200% from 2025 to 2050.^[2]^ Further improvements in the management of patients with AMI are urgently required to improve clinical outcome.^[2]^ Currently, primary percutaneous coronary intervention (PCI) is the gold standard treatment for patients with ST-segment elevation myocardial infarction (STEMI).^[3–5]^ Time to primary PCI is strongly associated with mortality risk of STEMI, and efforts to reduce door to balloon time will improve patient outcome.^[6]^ Accordingly, a door to balloon time of 90 minutes or less are recommended by current clinical guidelines on PCI.^[4,7]^ Importantly, recent studies have suggested that physician-led triage strategies may be effective in shortening door-to balloon time for patients with STEMI.^[8,9]^ In the present study, whether implementation of multidisciplinary collaboration strategies may further reduce door to wire time for STEMI patients undergoing PCI is investigated.

Objective: The aim of this study is to reduce door-to-wire time for ST-elevation myocardial infarction patients undergoing primary percutaneous coronary intervention through multidisciplinary collaboration.

## 2. Methods

Patients over the age of 18 who visited the Foshan Sanshui District People’s Hospital and were diagnosed with STEMI between 2018 and 2019 were included in this study. Patients who were diagnosed with in-hospital STEMI were excluded from this study.

Foshan Sanshui District People’s Hospital is a tertiary medical center in Southern China, serving a population of >800,000 people. Patients included in the study were segregated based on the date of presentation into a pre-intervention interim period in 2018 prior to the full implementation of intervention measures, and a post-intervention period in 2019. The data used in this study were collected from the China National Chest Pain Platform and hospital medical records.

## 3. Statistical analysis

Statistical analyses were performed using BM SPSS version 27 (IBM, Armonk). Results were presented as means with the corresponding standard deviations and compared by the Student *t* test, or reported as medians along with the inter-quartile range (IQR) where the nonparametric Mann–Whitney *U* test was utilized. The chi-square test and Fisher’s exact test were used for proportions. A *P* value of <.05 was considered statistically significant.

## 4. Results

### 4.1. Intervention measures

In attempt to shorten door to wire time, the following interventions were implemented:

1. Logistical organization and arrangements were made for all hospital employees to learn the relevant requirements, regulations and guidelines of the chest pain center allowing all staff to indirectly or directly contribute to STEMI recognition and facilitate subsequent care. Additional training sessions on recognizing chest pain as an urgent medical priority were also implemented for all new employees including those not directly involved in medical care such as cleaning staff, porters and security officers. Furthermore, regular training and teaching sessions were organized for staff in key departments of the chest pain center including the emergency department, cardiovascular medicine department, outpatient clinic, and catheterization laboratory to reinforce and further familiarize staff with the procedures for managing chest pain patients.
2. Increased media coverage were sought to improve public awareness of the emergency medicine telephone number 120 (in China). In AMI, coronary arteries should be recanalized within 120 minutes from chest pain in order to maximally salvage the necrotic myocardium. Therefore, the importance of calling 120 as soon as possible if chest pain occurs was highlighted in particular to the general public.
3. A Chest Pain Alliance WeChat work group was established to facilitate communication and improve time-to-diagnosis from patient presentation. All alliance hospital units, chest pain center staff, and electrocardiogram (ECG) expert doctors were added to the group, allowing chest pain cases to be triaged and diagnosed remotely wherever possible, and enabling treatment recommendations to be made with minimal delay.
4. Regular training sessions were provided to doctors and nurses in the emergency departments and outpatient clinics to improve confidence and accuracy in identifying myocardial infarction on ECGs and facilitate faster diagnosis.
5. Improvements were implemented to strengthen the logistics and staff training in Chest Pain Alliance Hospitals to shorten the entry-to-departure time of local hospitals to <30 minutes wherever feasible, thereby improving the door to wire time through faster transfer to PCI centers.
6. Conspicuous Chest Pain Center signs have been erected at the hospital entrance for patients with chest pain who come to the hospital on their own, and reminders to prioritize chest pain are placed throughout the emergency department. An ECG will be completed within 10 minutes after the first medical contact, and if STEMI is diagnosed, the catheterization lab will be notified immediately. At the same time, informed consent for PCI will be sought, and blood will be drawn to check troponin.
7. For patients with chest pain who have received prehospital ECGs and subsequently confirmed to be STEMI, the following process was implemented with the aim to shorten door to wire time: immediately call the chest pain center for patient transfer, activate the catheterization laboratory as per protocol, and start acquiring informed consent for the procedure wherever feasible.
8. For patients being transferred for PCI, the catheterization laboratory is activated as per protocol in the ambulance, and informed consent for the procedure is started.
9. Further training for emergency department and cardiovascular physicians on informed consent skills were provided, allowing them to explain the condition and the necessity of emergency PCI as quickly as possible to patients and families, and allowing them to make informed decisions.
10. Arrangements have been made for the catheterization laboratory to always have coronary surgery kits stocked, in order to avoid unnecessary delays upon patient arrival.
11. The Chest Pain Center has a designated full-time data clerk to summarize the timeline of each STEMI patient accurately. A running quality analysis meeting and a chest pain case discussion meeting are held every 2 months to analyze the causes of door to wire overtime cases and make improvements accordingly to reduce the number of breaches.
12. The Chest Pain Center telephone line is open 24 hours a day and is held by the chief resident of the Department of Cardiovascular Medicine to ensure immediate consultation, clear diagnosis and activation of the catheterization laboratory.
13. A special incentive mechanism was implemented to reward staff and improve morale for every door to wire case that met the standards.

Comparisons of the interim and post-intervention periods are depicted in Table 1. A total of 305 STEMI patients were initially identified in the study, with 5 excluded as they were in-hospital STEMI cases. The patients were classified into the interim period (2018) and post-intervention period (2019) based on date of presentation, and there were no significant differences in baseline characteristics including age, gender, atrial fibrillation, stroke history, hyperlipidemia, hypertension, diabetes and PCI history between the 2 groups. Overall, there was no difference in terms of in-hospital mortality between the 2 groups.

**Table 1:** Clinical comparison of interim and post-intervention periods.

	Interim period	Post-intervention period	*X*^2^/(*t*/*z*)	*P*
Amount	150	150		
Age mean ± SD	59.40 ± 12.64	58.73 ± 13.16	0.447	.655
Male gender, n (%)	129 (86.00)	121 (80.67)	1.536	.215
Atrial fibrillation, n (%)	6 (4.00)	4 (2.67)	0.414	.520
Prior stroke, n (%)	5 (3.33)	4 (2.67)	0.115	.735
Hyperlipemia, n (%)	86 (57.33)	89 (59.33)	0.123	.725
Hypertension, n (%)	54 (36.00)	57 (38.00)	0.129	.720
Diabetes, n (%)	30 (20.00)	43 (28.67)	3.060	.080
Prior PCI, n (%)	7 (4.67)	8 (5.33)	0.070	.791
In-hospital mortality, n (%)	1 (0.67)	3 (2.01)	1.027	.311

PCI = percutaneous coronary interventions, SD = standard deviation.

Time Trends of the interim and post-intervention periods are shown in Table 2. The median door to puncture time was reduced from 57.5 minutes in the interim period to 46.0 minutes (*P* ＜ .001) in post-intervention period. Similarly, median door to wire time was shortened from 88.0 minutes to 63.5 minutes (*P* ＜ .001). Strikingly, 24% of patients had a door to wire time of ≤60 minutes in the interim period, which is significantly lower than the 40.67% in the post-intervention period (*P* = .002).

**Table 2:** Time trends of interim and post-intervention periods.

	Interim period	Post-intervention period	*X*^2^/*z*	*P*
Median onset to door time, IQR (min)	145.5 (81.5, 284.3)	147.0 (81.5, 300.5)	−0.127	.899
Median onset to wire time IQR (min)	234.0 (171.8, 366.3)	224.5 (144.8, 369.3)	−1.314	.189
Median door to puncture time, IQR (min)	57.5 (38.5, 78.3)	46.0 (23.5, 61.0)	−3.753	<.001
Median door to wire time, IQR (min)	88.0 (60.0, 110.3)	63.5 (43.0, 82.5)	−5.485	<.001
Door to wire time ≤ 60 min, n (%)	36 (24.00)	61 (40.67)	9.522	.002
Door to wire time ≤ 90 min, n (%)	78 (52.00)	122 (81.33)	29.040	<.001

IQR = inter-quartile range.

## 5. Discussion

The present study demonstrates that door to wire time for STEMI patients undergoing PCI can be significantly reduced through the implementation of changes based on the principles of multidisciplinary collaboration.

Previous studies demonstrated that multidisciplinary collaboration and continuous process optimization can result in overall shortened door to needle time and door to puncture time for stroke patients.^[10–12]^ Similar to stroke,^[10]^ early treatment is crucial to improve outcome in AMI patients,^[13]^ and longer symptom to recanalization time is associated with increased infarct size and poor prognosis.^[13]^ Achieving shorter time to PCI for STEMI patients remains a critical challenge, hampered by inconsistent access to catheterization labs particularly during out-of-hours.^[14]^ Accordingly, the higher adjusted mortality rate associated with out-of-hours admissions for PCI can be partly explained by the longer reperfusion times.^[15,16]^

Clinical and logistical factors that also contribute to longer reperfusion time in STEMI patients include prehospital delay, atypical presentations without chest pain, and absence of classical ECG findings of STEMI.^[17]^ Transferred STEMI patients from another hospital face a longer time-to-PCI compared to those presenting directly to Chest Pain Centers with facilities for PCI, resulting in longer time to reperfusion and larger infarct sizes.^[18]^ Similarly, hemodynamic complications can result in longer time spent with the emergency medical services, further resulting in worse outcomes for STEMI patients. Emergency medical services delay time of STEMI patients should be closely monitored and reduced as appropriate.^[19]^

On the other hand, it is important to recognize that overly aggressive measures to reduce door to wire time may be associated with an increased risk of false-positive diagnosis of STEMI.^[20]^ Measures to reduce door to wire time should be monitored carefully to avoid unnecessary procedures, which are inherently associated with significant risks and in addition may result in the delay of more suitable therapies, especially for those that are critically ill.^[20]^ Overall, the door to wire time involves many factors and a multidisciplinary collaborative approach serves as an important strategy to reduce door to wire time for STEMI patients and may improve clinical outcome.

## 6. Limitation

Limitations of this study include those inherently associated with a retrospective single-center study and future prospective research is required.

## 7. Conclusion

“Time is muscle” – multidisciplinary collaboration is an important strategy to reduce door to wire time for STEMI patients. The intervention measures adopted in this study are effective in reducing door to wire time and may be implemented by suitable centers with the potential to improve AMI patient outcome.

## Acknowledgments

We would like to thank our colleagues for their help in data collection and care for patients. We also wish to thank all the departments enrolled in the study, and the patients for their invaluable contribution.

## Author contributions

**Conceptualization:** Xiaoru Zeng, Ling Chen, Francisco-Javier Roldan.

**Data curation:** Ling Chen.

**Formal analysis:** Jia chao Liu.

**Investigation:** Jianguang Liang.

**Methodology:** Eric Jou, Junrong Tu.

**Resources:** Guanglong Ma.

**Supervision:** Jianguang Liang, Zhen zhang Li.

**Visualization:** Xiaoru Zeng, Ayush Chandra.

**Writing – original draft:** Xiaoru Zeng, Xiaodong Zheng, Wanling Pan, Wanquan Li.

**Writing – review & editing:** Ayush Chandra, Xiaodong Zheng, Shengde Xie, Francisco-Javier Roldan, Zhen zhang Li.

## References

[1] Reed GW Rossi JE Cannon CP. Acute myocardial infarction. Lancet. 2017;389:197–210. 27502078 10.1016/S0140-6736(16)30677-8

[2] Xu H Yang Y Wang C. China Acute Myocardial Infarction Registry Investigators. Association of hospital-level differences in care with outcomes among patients with acute ST-segment elevation myocardial infarction in China. JAMA Netw Open. 2020;3:e2021677. 33095249 10.1001/jamanetworkopen.2020.21677 PMC7584928

[3] Lambert L Brown K Segal E Brophy J Rodes-Cabau J Bogaty P. Association between timeliness of reperfusion therapy and clinical outcomes in ST-elevation myocardial infarction. JAMA. 2010;303:2148–55. 20516415 10.1001/jama.2010.712

[4] Ibanez B James S Agewall S. 2017 ESC Guidelines for the management of acute myocardial infarction in patients presenting with ST-segment elevation: the task force for the management of acute myocardial infarction in patients presenting with ST-segment elevation of the European Society of Cardiology (ESC). Eur Heart J. 2018;39:119–77. 28886621 10.1093/eurheartj/ehx393

[5] Karabağ Y Çağdaş M Rencuzogullari I. Comparison of SYNTAX score II efficacy with SYNTAX score and TIMI risk score for predicting in-hospital and long-term mortality in patients with ST segment elevation myocardial infarction. Int J Cardiovasc Imaging. 2018;34:1165–75. 29541904 10.1007/s10554-018-1333-1

[6] McNamara RL Wang Y Herrin J. NRMI Investigators. Effect of door-to-balloon time on mortality in patients with ST-segment elevation myocardial infarction. J Am Coll Cardiol. 2006;47:2180–6. 16750682 10.1016/j.jacc.2005.12.072

[7] O’Gara PT Kushner FG Ascheim DD. 2013 ACCF/AHA guideline for the management of ST-elevation myocardial infarction: executive summary: a report of the American College of Cardiology Foundation/American Heart Association Task Force on Practice Guidelines. J Am Coll Cardiol. 2013;61:485–510. 23256913 10.1016/j.jacc.2012.11.018

[8] Karakayali M Omar T Artac I. The prognostic value of HALP score in predicting in-hospital mortality in patients with ST-elevation myocardial infarction undergoing primary percutaneous coronary intervention. Coron Artery Dis. 2023;34:483–8. 37799045 10.1097/MCA.0000000000001271

[9] Schwarzfuchs D Shashar S Sagy I Novack V Zeldetz V. Does the physician in triage strategy improve door-to-balloon time for patients with STEMI? Emerg Med J. 2020;37:540–5. 32753394 10.1136/emermed-2019-209241

[10] Chen Y Nguyen TN Wellington J. Shortening door-to-needle time by multidisciplinary collaboration and workflow optimization during the COVID-19 pandemic. J Stroke Cerebrovasc Dis. 2022;31:106179. 34735901 10.1016/j.jstrokecerebrovasdis.2021.106179 PMC8526426

[11] Yang S Yao W Siegler JE. Shortening door-to-puncture time and improving patient outcome with workflow optimization in patients with acute ischemic stroke associated with large vessel occlusion. BMC Emerg Med. 2022;22:136. 35883030 10.1186/s12873-022-00692-8 PMC9315077

[12] Cheng W Mofatteh M Baizabal-Carvallo JF. Impact of thrombolysis time metrics when participating in national stroke center construction project. J Multidiscip Healthc. 2023;16:3333–8. 37954470 10.2147/JMDH.S432458 PMC10638894

[13] Greulich S Mayr A Gloekler S. Time-dependent myocardial necrosis in patients with ST-segment-elevation myocardial infarction without angiographic collateral flow visualized by cardiac magnetic resonance imaging: results from the multicenter STEMI-SCAR project. J Am Heart Assoc. 2019;8:e12429. 10.1161/JAHA.119.012429 PMC6645633 31181983

[14] Casella G Ottani F Ortolani P. Off-hour primary percutaneous coronary angioplasty does not affect outcome of patients with ST-segment elevation acute myocardial infarction treated within a regional network for reperfusion: the REAL (Registro Regionale Angioplastiche dell’Emilia-Romagna) registry. JACC Cardiovasc Interv. 2011;4:270–8. 21435603 10.1016/j.jcin.2010.11.012

[15] Jayawardana S Salas-Vega S Cornehl F Krumholz HM Mossialos E. The relationship between off-hours admissions for primary percutaneous coronary intervention, door-to-balloon time and mortality for patients with ST-elevation myocardial infarction in England: a registry-based prospective national cohort study. BMJ Qual Saf. 2020;29:541–9. 10.1136/bmjqs-2019-010067 PMC7362773 31831635

[16] Sorita A Ahmed A Starr SR. Off-hour presentation and outcomes in patients with acute myocardial infarction: systematic review and meta-analysis. BMJ. 2014;348:f7393. 24452368 10.1136/bmj.f7393 PMC3898160

[17] Peterson MC Syndergaard T Bowler J Doxey R. A systematic review of factors predicting door to balloon time in ST-segment elevation myocardial infarction treated with percutaneous intervention. Int J Cardiol. 2012;157:8–23. 21757243 10.1016/j.ijcard.2011.06.042

[18] Forsyth R Sun ZH Reid C Moorin R. Inter-hospital transfers and door-to-balloon times for STEMI: a single centre cohort study. J Geriatr Cardiol. 2020;17:321–9. 32670362 10.11909/j.issn.1671-5411.2020.06.001 PMC7338936

[19] Alrawashdeh A Nehme Z Williams B. Impact of emergency medical service delays on time to reperfusion and mortality in STEMI. Open Heart. 2021;8:e001654. 33963080 10.1136/openhrt-2021-001654 PMC8108686

[20] Fanari Z Abraham N Kolm P. Aggressive measures to decrease “door to balloon” time and incidence of unnecessary cardiac catheterization: potential risks and role of quality improvement. Mayo Clin Proc. 2015;90:1614–22. 26549506 10.1016/j.mayocp.2015.08.021 PMC4679675

